# THERANAP: AN EDUCATIONAL SLEEP HEALTH INTERVENTION FOR FRAIL OLDER ADULTS

**DOI:** 10.1093/geroni/igad104.3670

**Published:** 2023-12-21

**Authors:** Amy Berkley

**Affiliations:** St. Louis University, St. Louis, Missouri, United States

## Abstract

Napping and other daytime sleep is often overlooked and poorly defined in insomnia research. Studies have shown correlations between older adults’ napping habits and increased medical co-morbidities and risks of dementia, but others report napping enhances memory consolidation and broader aspects of cognition in younger adults. Why is napping recommended for younger adults but considered problematic for older adults? In previous a research study, (18 older adults in an independent living facility: 6 men,12 women, mean age 84, SD= 7.62, range 67-96, who self-reported sleep problems) participants reported napping in qualitative interviews but denied daytime sleep on standard sleep assessments. Participants spoke about napping apologetically and expressed dread of its links to functional and cognitive decline. They stigmatized planned napping, which could potentially benefit their cognitive functioning, but seemed accepting of accidental napping, which could well indicate more serious physical or cognitive issues To address these issues, TheraNap teaches intentional napping as a therapy. This sleep health education intervention empowers sleep-deprived older adults to take control of their sleep habits by practicing intentional napping to improve overall sleep health. TheraNap includes: • Short sleep health training session with recommended napping guidelines • Two-week long planned napping routines • A sleep diary • Supplementary sleep meditations • Follow-session to assess perceptions of sleep health More research is needed into the ways napping can improve physical and cognitive function, and more education is needed for older adults, their families and caregivers, and LTC staff about the importance of sleep for older adults.

